# Multimorbidity and the risk of depression: a systematic review of cohort studies

**DOI:** 10.7189/jogh.16.04279

**Published:** 2026-09-25

**Authors:** Jingjing Wu, Keying Xu, Ping Jiang, Hua Mei

**Affiliations:** 1 School of Postgraduate Studies, Shanghai University of Traditional Chinese Medicine, Shanghai, China; 2 School of Nursing and Health Management, Shanghai University of Medicine and Health Sciences, Shanghai, China; 3 Department of Nursing, Pudong New Area People's Hospital, Shanghai, China

**Keywords:** multimorbidity, multimorbidity patterns, depression, cohort study, systematic review

## Abstract

**Background:**

Multimorbidity has been associated with an increased risk of depression. In this systematic review, we aimed to evaluate longitudinal evidence on the association between multimorbidity, including specific multimorbidity patterns, and the risk of developing depression.

**Methods:**

We searched Web of Science, PubMed, Embase, PsycINFO, China National Knowledge Infrastructure, and Wanfang from database inception to 31 December 2025. Cohort studies examining the association between multimorbidity or multimorbidity patterns and incident depression or depressive symptoms were included. Two reviewers independently screened studies, extracted data, and assessed study quality using the Newcastle–Ottawa Scale. We employed a narrative synthesis approach and presented the results using descriptive forest plots and harvest plots.

**Results:**

A total of 17 longitudinal cohort studies were included, conducted in accordance with PRISMA guidelines, with generally high overall quality. Multimorbidity was consistently associated with an increased risk of depression, and the risk generally increased with the number of chronic conditions. This association was relatively stable across genders and populations in different countries. Associations varied across multimorbidity patterns. Cardiovascular-metabolic and respiratory-related patterns showed the most consistent associations with depression, whereas evidence for other patterns was limited and less consistent. Cardiovascular-metabolic multimorbidity showed a cumulative effect. Increasing multimorbidity trajectories may also be associated with subsequent depression risk.

**Conclusions:**

Multimorbidity was longitudinally associated with an increased risk of incident depression or depressive symptoms, and this association appeared to vary according to disease burden, multimorbidity patterns, and changes in multimorbidity over time. Cardiovascular-metabolic patterns, respiratory-related patterns, and rapid trajectories of multimorbidity may represent potential high-risk features requiring further validation. Future research should standardise definitions and pattern identification methods, and further investigate dynamic processes and underlying mechanisms to inform early identification, risk stratification, and future intervention development for depression.

**Registration:**

PROSPERO: CRD420261302076

Multimorbidity generally refers to the coexistence of two or more chronic conditions in the same individual [1]. Its prevalence continues to rise with population ageing, prolonged survival of chronic non-communicable diseases, and the long-term accumulation of risk factors. A recent meta-analysis showed that the pooled prevalence of multimorbidity among older adults worldwide has reached 46% [2], exceeding 50% in most high-income countries. At the same time, the clinical and public health implications of multimorbidity are becoming increasingly apparent. Multimorbidity is associated with a range of adverse health outcomes, including accelerated physical decline, reduced health-related quality of life, and increased healthcare resource utilisation and costs [3].

Depression constitutes a significant component of the global burden of disease and is also a major component of multimorbidity. According to data from the World Health Organization, approximately 5% of adults worldwide suffer from depressive disorders, affecting around 280 million people [4]. Depression accounts for 4.46% of global disability-adjusted life years and 12.1% of years lived with disability [5]. The prevalence of depression is notably higher among individuals with multimorbidity than in the general population. In China, multimorbidity is associated with a 36% increased risk of depression, while the association is even more pronounced in the USA (risk ratio (RR) = 1.61) [6]. In India, the prevalence of depression among older adults with multimorbidity is close to 29% [7]. A systematic review further reported that the prevalence of co-occurring depression reached 46.7% among individuals aged ≥60 years with multimorbidity [8]. In addition, individuals with multimorbidity have a significantly higher risk of developing depression compared with those without chronic conditions [9]. Chronic conditions such as cardiovascular disease and diabetes are associated with an increased risk of depression, while depression may in turn contribute to disease progression through adverse health behaviours, forming a vicious cycle [10]. Furthermore, the co-occurrence of depression and multimorbidity is associated with reduced treatment adherence, increased symptom burden, accelerated disease progression, and a higher risk of all-cause mortality [11].

Current research on multimorbidity and depression mainly focuses on several areas. The first category describes prevalence and associations. These studies examine the prevalence of depression among individuals with multimorbidity across different populations and its relationship with the number of chronic conditions. Overall, they suggest that a higher level of multimorbidity is associated with a greater risk of depression [12]. The second category investigates the association between specific chronic diseases or multimorbidity and depression. This includes individual conditions such as cardiovascular disease, diabetes, stroke, and chronic pain, as well as multimorbidity defined as ‘≥2 chronic conditions’ or ‘≥3 chronic conditions’ [13]. The third category has gradually shifted towards multimorbidity patterns. These studies identify disease combinations, such as cardio-metabolic and neuropsychiatric patterns, and examine their differential associations with depression [14]. The fourth category focuses on the impact of depression in individuals with multimorbidity, examining outcomes such as functional impairment, reduced quality of life, increased healthcare utilisation, and mortality risk [15]. In addition, some studies have explored potential underlying mechanisms, including biological pathways such as chronic inflammation and vascular burden, and psychosocial pathways such as activity limitation and chronic pain [16].

Although existing research generally supports the association between multimorbidity and depression, several key limitations remain. First, most studies focus on describing the prevalence of depression or predicting risk [17]. There is limited systematic evidence on the possible pathways linking multimorbidity with subsequent depression. Second, the current evidence is mainly based on cross-sectional studies, making it difficult to clarify the temporal sequence between multiple conditions and depression [9]. Palmesed *et al.* conducted a systematic review on the impact of specific multimorbidity patterns on mental health outcomes [14]. However, their focus on depressive outcomes was limited. Longitudinal cohort studies can better characterise the dynamic relationship between multimorbidity and the onset of depression over time. However, the systematic integration of findings from cohort studies remains limited. Therefore, we sought to systematically evaluate the association between multimorbidity, including specific multimorbidity patterns, and the risk of depression based on cohort evidence. We aimed to provide longitudinal evidence that may help identify populations at potentially higher risk of depression and inform future research on risk stratification and intervention development.

## METHODS

We conducted and reported this systematic review in accordance with the PRISMA 2020 guidelines [18] (Table S1 in the **Online Supplementary Document**). The study protocol was prospectively registered in PROSPERO (registration number: CRD420261302076).

### Literature search strategy

We conducted a systematic literature search across six major databases: Web of Science, PubMed, Embase, PsycINFO, China National Knowledge Infrastructure, and Wanfang Database. We aimed to identify cohort studies on the association between multimorbidity and the risk of depression. The search covered the period from the inception of each database to 31 December 2025, and the final search was conducted on 5 March 2026. We used a combination of subject headings and free-text terms (Table S2 in the **Online Supplementary Document**). We also reviewed the reference lists of all included studies to identify any relevant studies that may have been missed.

### Inclusion and exclusion criteria

We included studies that investigated the association between multimorbidity or specific multimorbidity patterns and the risk of developing depression; studies that reported risk estimates, including adjusted RRs, hazard ratios (HRs), or odds ratios, along with their 95% confidence intervals (CIs); and peer-reviewed studies published in Chinese or English. We excluded non-human studies; non-adult populations or non-longitudinal cohort designs; non-original research (*e.g.* reviews, meta-analyses, conference abstracts, editorials, letters, or reports); case-control studies or randomised controlled trials; and studies that did not clearly define multimorbidity, or that only counted the number of symptoms, infectious diseases, or disease severity indices rather than the number or patterns of chronic conditions.

### Literature screening and data extraction

Two researchers (JW and KX) independently conducted the literature search and data extraction. Any disagreements were resolved through consultation with a third reviewer (HM), who also verified the accuracy of the extracted data. The following information was extracted from the included studies: first author, year of publication, country, data source, sample size, participant characteristics (age and gender), follow-up duration, number of diseases included, method of multimorbidity assessment, method of multimorbidity pattern identification, depression assessment or diagnosis, covariates, and main conclusions.

### Assessment of study quality

Two reviewers (JW and KX) used the Newcastle–Ottawa Quality Assessment Scale [19] to assess the quality of the included cohort studies (Table S3 in the **Online Supplementary Document**), with a third reviewer (HM) resolving any assessment discrepancies. The Newcastle–Ottawa Scale assigns a maximum of nine points across three domains: study selection (0–4 points), comparability of groups (0–2 points), and outcome assessment (0–3 points). Studies were classified as low (0–3 points), moderate (4–6 points), or high quality (7–9 points).

### Evaluation method

Given the substantial clinical and methodological heterogeneity across the included studies, a quantitative meta-analysis was considered inappropriate. Specifically, studies differed considerably in the definition of multimorbidity and the number of chronic conditions included (ranging from 7 to 69 conditions), methods used to identify multimorbidity patterns, depression assessment approaches, effect measures reported (*e.g.* HRs, RRs, odds ratios, and other effect estimates), and reference groups used for comparison. In addition, multimorbidity patterns varied substantially in their composition across studies, limiting the comparability of pooled estimates. Therefore, we conducted a narrative synthesis in accordance with the Synthesis Without Meta-analysis guideline. We used descriptive forest plots to visualise the main study findings [20]. Due to substantial variation in the definitions and composition of multimorbidity patterns across studies, we used a pattern harmonisation procedure (Table S4 in the **Online Supplementary Document**). We classified patterns reported in the original studies according to their dominant disease systems and mapped them into a set of standardised categories. For patterns involving multiple disease systems, classification was based on the dominant disease system or overall clinical profile described in the original study. We used forest plots to present the direction and distribution of associations between multimorbidity patterns and depression, with bar height representing the corresponding study sample size.

We used R software, version 4.4.2 (R Core Team, Vienna, Austria) for all analyses.

## RESULTS

### Literature search and study characteristics

**Figure 1:**
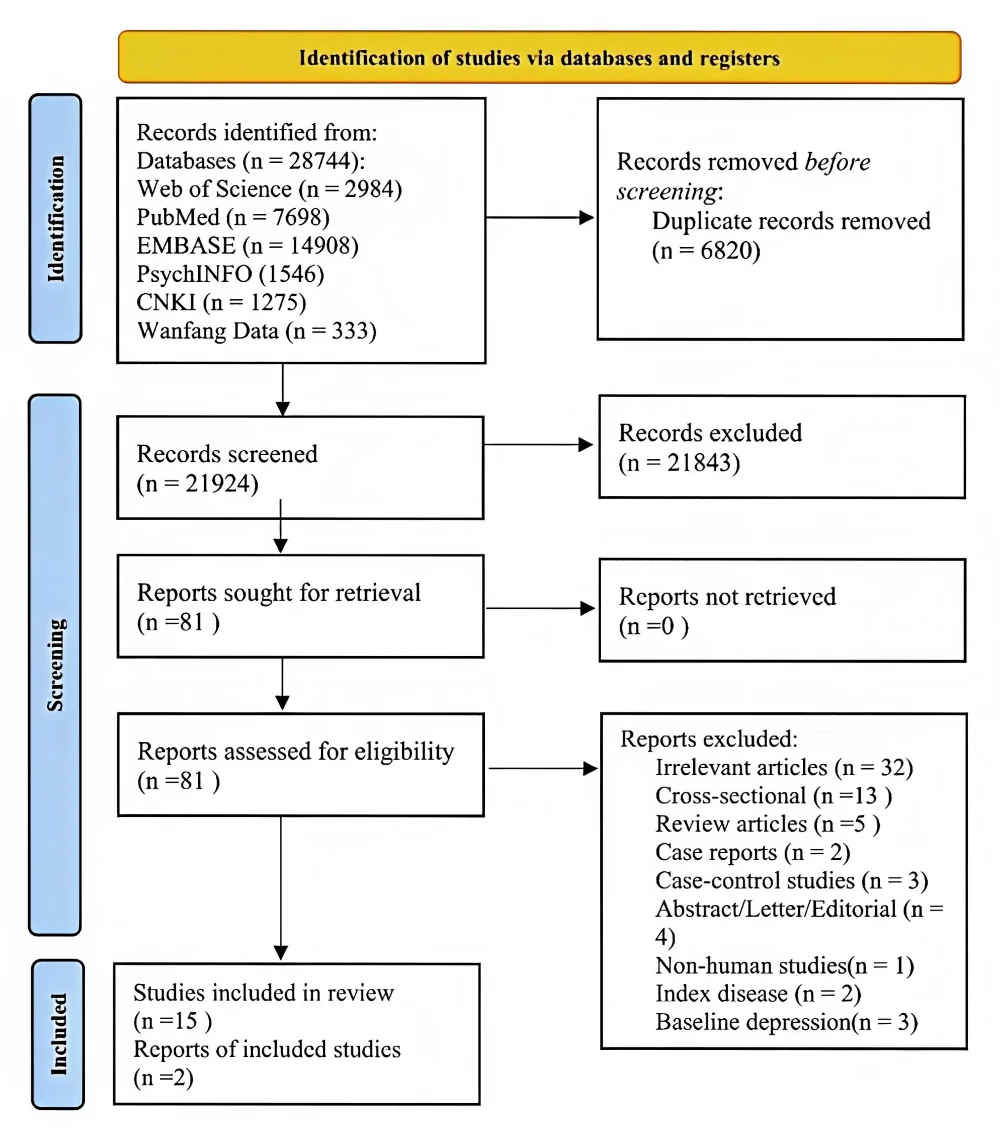
Flowchart of the literature search process.

An initial database search identified 28,744 records. After removing duplicates and screening titles and abstracts, 81 full-text articles were assessed. Based on the eligibility criteria, 17 studies were ultimately included (**Figure 1**, **Table 1**).

**Table 1:** Characteristics of included studies

Study, year, reference	Country; database/setting	Sample size (female %)	Average age, x̄ (SD)	MM numbers & assessment/clustering methods	Follow-up length in years	Depression assessment/measurement (effect size)	Adjusted covariates	Main findings (effect estimates)


Hsu *et al.* (2013) [21]

Taiwan, China; TLSA

4764 (46.2)

65.7 (9.2)

Number of diseases in MM: 10; MM assessment: self-report physician diagnosis; Method to identify MM cluster: frequency

11

CESD-10 (*β*)

Age, gender, years of formal education

MM patterns (N diseases ref): chronic respiratory disease (*β* = 1.332, *P* < 0.05); CVD (*β* = 1.160, *P* < 0.001); gastrointestinal disease (*β* = 1.200, *P* < 0.01); cancer (*β* = −0.282); CRD  + CVD (*β* = 2.802, *P* < 0.001); CRD + GI (*β* = 3.829, *P* < 0.01); CRD + cancer (*β* = 0.581); CVD + GI (*β* = 2.119, *P* < 0.001); CVD + cancer (*β* = 2.119, *P* < 0.001); GI + cancer (*β* = −7.499)

Ronaldson *et al.* (2021) [22]

UK; UK Biobank

154,367 (56.5)

57 (50–62)*

Number of diseases in MM: 36; MM assessment: self-report lifetime diagnoses + hospital admission records (HES); Method to identify MM cluster: EFA

7.6

PHQ-9 (HR)

Age, gender, ethnicity, social deprivation, education level, employment status, alcohol intake, smoking, physical activity, BMI, levels of CRP

Risk of depression: 0–1 *vs.* 2 (OR = 1.41; 95% CI = 1.32–1.53); 0–1 *vs.* 3 (OR = 1.94; 95% CI = 1.76–2.14); 0–1 *vs.* 4 (OR = 2.38; 95% CI = 2.07–2.74); 0–1 *vs.* ≥5 (OR = 2.89; 95% CI = 2.42–3.45); MM patterns (N MM ref): cardiometabolic patterns (HR = 1.93; 95% CI = 1.68–2.23); respiratory patterns (HR = 3.23; 95% CI = 2.44–4.27); cardio/cerebrovascular patterns (HR = 2.11; 95% CI = 1.45–3.07); reproductive pattern (HR = 2.04; 95% CI = 1.70–2.46); pain/gastrointestinal pattern (HR = 2.19; 95% CI = 1.92–2.50)

Ye *et al.* (2022) [23]

China; CHARLS

4605 (48.1)

≥45

Number of diseases in MM: 12; MM assessment: Self-reported physician diagnosis

4

CESD-10 (OR)

Age, gender, marital status, residential region, education level, BMI, per capita expenditure

Risk of depression: 0–1 *vs. *≥2 (OR = 2.05; 95% CI = 1.71–2.46); 0–1 *vs.* 2 (OR = 1.88; 95% CI = 1.52–2.34; 0–1 *vs.* 3 (OR = 2.05; 95% CI = 1.52–2.76); 0–1 *vs. *≥4 (OR = 2.82; 95% CI = 1.97–4.03)

Qiao *et al.* (2021) [24]

China; CHARLS

7587 (53.8)

≥45

Number of diseases in MM: 13; MM assessment: Self-reported physician diagnosis

4

CESD-10 (RR)

Age, gender, marital status, place of residence, BMI, education, smoking status, drinking

Risk of depression: 0 *vs.* 1 (RR = 1.08; 95% CI = 0.96–1.22); 0 *vs.* 2 (RR = 1.39; 95% CI = 1.22–1.57); 0 *vs.* 3 (RR = 1.46; 95% CI = 1.23–1.70); 0 *vs.* ≥4 (RR = 1.62; 95% CI = 1.34–1.93)

Yao *et al.* (2020) [25]

China; CHARLS

10,084 (46.7)

57.7 (NA)

Number of diseases in MM: 16; MM assessment: Self-reported physician diagnosis; Method to identify MM cluster: EFA

4

CESD-10 (OR)

Age, sex, education, residence, marital status, smoking status, frequency of alcohol consumption, social participation in the last month, BMI

Risk of depression: 0 *vs.* 1 (OR = 1.21; 95% CI = 1.04–1.40); 0 *vs.* 2 (OR = 1.66; 95% CI = 1.40–1.98); 0 *vs.* ≥3 (OR = 2.11; 95% CI = 1.74–2.57); MM patterns (lowest factor scores of each MM pattern ref): cardio-metabolic pattern (OR = 1.12; 95% CI = 1.06–1.20); respiratory pattern (OR = 1.25; 95% CI = 1.17–1.33); arthritic-digestive-visual pattern (OR = 1.29; 95% CI = 1.22–1.37); hepatic-renal-skeletal pattern (OR = 1.09; 95% CI = 1.02–1.16)

Zhou *et al.* (2023) [26]

Canada; CLSA

7163 (47)

58.4 (9.0)

Number of diseases in MM: 13; MM assessment: Self-reported physician diagnosis

4

CESD-10 (HR)

Age, gender, the highest level of education, marital status, health insurance status, nighttime sleep duration, drinking status, smoking status, social activity participation, income status, BMI, region

Risk of depression: 0 *vs.* 1 (HR = 1.197; 95% CI = 1.096–1.308); 0 *vs.* 2 (HR = 1.310; 95% CI = 1.180–1.454); 0 *vs.* ≥3 (HR = 1.397; 95% CI = 1.241–1.572)

Guo *et al.* (2025) [27]

China; CHARLS

6455 (48.67)

≥45

Number of diseases in MM: 5; MM assessment: self-report physician diagnosis; clinical measurements

7

CESD-10 (HR)

Age, sex, marital status, poor childhood health, existing disability, mental integrity, smoking, alcohol consumption, SES

Risk of depression: 0 *vs.* 1 (HR = 1.08; 95% CI = 1.00–1.19); 0 *vs.* ≥2 (HR = 1.13; 95% CI = 1.02–1.26); 0 *vs.* 1 (HR = 1.01; 95% CI = 0.89–1.13; age 45–59) and (HR = 1.26; 95% CI = 1.09–1.45; age ≥60); 0 *vs.* ≥2 (HR = 1.06; 95% CI = 0.91–1.23; age 45–59) and (HR = 1.30; 95% CI = 1.12–1.52; age ≥60)

Du *et al.* (2025) [12]

China; MHAS, KLoSA, SHARE, HRS, CHARLS, ELSA

23,947 (51.7)

≥65

Number of diseases in MM: 7; MM assessment: self-report physician diagnosis

13

CESD-10/EURO-D (HR)

Age, gender, marital status, number of people living in the household, educational level, total household income, BMI, smoking status, alcohol consumption, physical activity, functional impairment factors

Risk of depression: 0–1 *vs. *≥2 (HR = 1.30; 95% CI = 1.25–1.37); 0 *vs.* 1 (HR = 1.15; 95% CI = 1.09–1.211); 0 *vs.* 2 (HR = 1.37; 95% CI = 1.29–1.45); 0 *vs. *≥3 (HR = 1.57; 95% CI = 1.45–1.70); female 0–1 *vs.* ≥2 (HR = 1.31; 95% CI = 1.25–1.37); male 0–1 *vs. *≥2 (HR = 1.40; 95% CI = 1.30–1.51); 65–74 years (HR = 1.36; 95% CI = 1.28–1.44); ≥75 years (HR = 1.22; 95% CI = 1.13–1.32)

Ho *et al.* (2023) [28]

Taiwan, China; TLSA

1975 (48.7)

62.1 (7.6)

Number of diseases in MM: 12; MM assessment: Self-reported physician diagnosis; Method to identify MM cluster: LCA

15

CESD-10 (OR)

Age, sex, income level, social participation, self-rated health, health behaviour, disability, admission

MM patterns (relatively healthy cluster ref): cardiometabolic c pattern (OR = 1.33; 95% CI = 0.745–2.375); arthritis-cataract cluster (OR = 1.23; 95% CI = 0.704–2.147); MM cluster: (OR = 1.623; 95% CI = 1.019–2.584)

Triolo *et al.* (2024) [29]

Sweden; SNAC-K

2904 (63.1)

73.2 (10.5)

Number of diseases in MM: 54; MM assessment: Clinical assessment, medication review, and national registers; Method to identify MM cluster: LCA

9.6

CPRS (HR)

Age, sex, education, civil status, alcohol consumption, smoking, malnutrition

MM patterns (none multimorbid cluster ref): metabolic pattern (HR = 1.06; 95% CI = 0.53 − 2.15); sensory/anaemia pattern (HR = 1.91; 95% CI = 1.03–3.53); thyroid/musculoskeletal pattern (HR = 1.90; 95% CI = 1.06–3.39); cardiometabolic pattern: (HR = 2.77; 95% CI = 1.40–5.46)

Zhang *et al.* (2025) [30]

China; CHARLS

1988 (44.3)

67 (NA)

Number of diseases in MM: 12; MM assessment: Self-reported physician diagnosis; Method to identify MM cluster: LCA

9.17

CESD-10 (HR)

Age, sex, education, residence, marital status, smoking status, frequency of alcohol consumption, social participation, BMI

MM patterns (relatively healthy pattern ref): metabolic syndrome/vascular pattern (HR = 1.08; 95% CI = 0.89–1.32); digestion/arthritis pattern (HR = 1.47; 95% CI = 1.22–1.77); respiratory pattern (HR = 1.47; 95% CI = 1.07–2.04)

Wister *et al.* (2023) [31]

Canada; CLSA

18,099 (51.56)

>65

Number of diseases in MM: 27; MM assessment: self-reported physician diagnosis; Method to identify MM cluster: predetermined method

3.5

CESD-10 (OR)

Sex, age, education, country of birth, ethnicity, marital status, personal income, household size, working status, living area

MM patterns (N MM ref) Cardio pattern (OR = 1.35; 95% CI = 1.08–1.63); respiratory pattern (OR = 2.76; 95% CI = 1.91–3.60); osteo pattern (OR = 1.67; 95% CI = 1.38–1.96); cancer (OR = 0.86; 95% CI = 0.59–1.12); multiple patterns (OR = 2.09; 95% CI = 1.82–2.36); others (OR = 1.07; 95% CI = 0.85–1.20)

DeLong *et al.* (2025) [32]

UK; UK Biobank

140,956 (51.81)

NA

Number of diseases in MM: 69; MM assessment: EHR-based diagnoses; Method to identify MM cluster: *k*-modes clustering analysis

6.8

Diagnoses, hospital records, cancer registry records (HR)

Age, sex, ethnicity, country of residence, deprivation

MM clusters (health group ref): respiratory cluster (HR = 1.95; 95% CI = 1.74–2.18); CVD + Diabetes cluster: (HR = 1.78; 95% CI = 1.61–1.97); mixed including cancer (HR = 1.62; 95% CI = 1.48–1.77); healthy + rhinitis (HR = 1.59; 95% CI = 1.46–1.75); women respiratory cluster (HR = 1.93; 95% CI = 1.67–2.23); musculoskeletal cluster: (HR = 1.81; 95% CI = 1.58–2.07); CVD + diabetes cluster (HR = 1.71; 95% CI = 1.52–1.94); mixed including cancer (HR = 1.63; 95% CI = 1.46–1.82); healthy + rhinitis (HR = 1.48; 95% CI = 1.30–1.67); Men: Musculoskeletal + others (HR = 2.50; 95% CI = 2.15–2.91); respiratory + gout + male genitourinary (HR = 2.25; 95% CI = 1.43–3.53); CVD + diabetes (HR = 1.90; 95% CI = 1.65– 2.20); mixed including cancer (HR = 1.60; 95% CI = 1.38–1.86); healthy + rhinitis (HR = 1.50; 95% CI = 1.29–1.75)

Yang *et al.* (2024) [33]

China; STR

40,080 (53.3)

60.47 (10.59)

Number of diseases in CMM: 3; MM assessment: self-report physician diagnosis

18

Medical records (HR)

Age, sex, education, marital status, smoking status, alcohol consumption, physical activity level, BMI, hypertension

Risk of depression (N CMDs ref): CMD MM (HR = 1.83; 95% CI = 1.29–2.58); stroke (HR = 1.31; 95% CI = 0.87–1.99); heart disease (HR = 1.71; 95% CI = 1.43–2.04); diabetes (HR = 1.29; 95% CI = 1.01–1.66); diabetes + heart disease (HR = 1.75; 95% CI = 1.10–2.80); heart disease + stroke (HR = 2.44; 95% CI = 1.34–4.43); diabetes + stroke (HR = 1.17; 95% CI = 0.38–3.64); diabetes + heart disease + stroke (HR = 1.61; 95% CI = 0.40–6.44)

Huang *et al.* (2022) [34]

China; CHARLS

18,002 (51)

56 (49–63)*

Number of diseases inC MM: 5; MM assessment: Self-reported physician diagnosis + clinical measurements

7

CESD-10 (OR)

Age, sex, residential region, educational level, marital status, smoking status, alcohol consumption, BMI, other chronic diseases

Risk of depression (N CMDs ref): CMD MM (OR = 1.59; 95% CI = 1.42–1.78); stroke (OR = 1.62; 95% CI = 1.40–1.88); heart disease (OR = 1.30; 95% CI = 1.19–1.42); diabetes (OR = 1.14; 95% CI = 1.05–1.24); specific combination heart disease + diabetes (OR = 1.32; 95% CI = 1.02–1.70); stroke + hypertension (OR = 1.49; 95% CI = 1.15–1.94); diabetes + hypertension (OR = 1.20; 95% CI = 1.05–1.38); heart disease + hypertension (OR = 1.34; 95% CI = 1.17–1.54); heart disease + diabetes + dyslipidaemia (OR = 1.60; 95% CI = 1.18–2.18)

Zhou *et al.* (2024) [11]

China; CHARLS, KLoSA, ELSA, HRS, SHARE

67,188 (52.5)

64.36(9.22)

Number of diseases in CMM: 3; MM assessment: Self-reported

7

CESD-10/CESD-8/EURO-D12 (HR)

Age, sex, marital status, educational level, total household wealth, smoking status, drinking status, physical activity, BMI, chronic conditions

Risk of depression (N CMDs ref): CMD MM (HR = 1.40; 95% CI = 1.31–1.50); diabetes (HR = 1.20; 95% CI = 1.13–1.26); heart diseases (HR = 1.26; 95% CI = 1.19–1.33); stroke (HR = 1.31; 95% CI = 1.19–1.46); diabetes + heart diseases (HR = 1.50; 95% CI = 1.37–1.64); diabetes + stroke: (HR = 1.58; 95% CI = 1.32–1.89); heart diseases + stroke (HR = 1.50; 95% CI = 1.31–1.72); diabetes + heart diseases + stroke (HR = 1.60; 95% CI = 1.32–1.94)

Zhang *et al.* (2025) [35]

China; CHARLS

15,896 (52.7)

NA

Number of diseases in CMM: 14; MM assessment: self-report physician diagnosis

9

CESD-10 (OR)

Age, gender, residency status, marital status, SES, smoking, alcohol consumption, physical activity

Risk of depression (0–1 *vs.*≥2) (OR = 2.36; 95% CI = 2.24–2.49); MM trajectories (no new condition ref): slow growth (OR = 1.53; 95% CI = 1.36–1,71); steady growth (OR = 2.54; 95% CI = 2.24–2.89); rapid growth (OR = 4.40; 95% CI = 3.62–5.34)

BMI – body mass index, CESD-10 – 10 items of Center for Epidemiologic Studies of Depression Scale, CESD-8 – 8 items of Center for Epidemiologic Studies of Depression Scale, CHARLS – China Health and Retirement Longitudinal Study, CI – confidence interval, CMDs – cardiometabolic diseases, CMM – cardiometabolic multimorbidity, CPRS – Computerised Patient Record System, CRP – C-reactive protein, CRD – chronic respiratory disease, CVD – cardiovascular disease, EFA – exploratory factor analysis, ELSA – the English Longitudinal Study of Ageing, EURO-D12 – 12-item version of the EURO-D, HR – hazard ratio, HRS – Health and Retirement Study, IRR – incident rate ratio, KLoSA – Korean Longitudinal Study of Aging, LCA – latent class analysis, MHAS – Mexican Health and Aging Study, MM – multimorbidity, NA – not available, OR – odds ratio, PHQ-9 – 9 items of Patient Health Questionnaire, ref – reference, RR – risk ratios, SD – standard deviation, SES – socioeconomic status, SHARE – Survey of Health, Ageing and Retirement in Europe, SNAC-K – Swedish National Study on Aging and Care in Kungsholmen, STR – Swedish Twin Registry, TLSA – Taiwan Longitudinal Study on Aging, x̄ – mean

*Values expressed as median (interquartile range).

A total of 15 studies were published within the last five years. Nearly half used individual-level data from the China Health and Retirement Longitudinal Study (CHARLS) [12,23–25,27,30,34,35], while the remaining studies were mainly conducted in European countries using databases such as the UK Biobank, Canadian Longitudinal Study on Aging, and Swedish Twin Registry. The sample size ranged from 1975 to 154,367 participants, with a total sample exceeding 500,000. Most studies (n = 11, 65%) included populations with an average age of ≥60 years. Some studies also included middle-aged participants aged ≥45 years [11,12,23–25,27,34]. The proportion of women varied across studies, with most reporting a female proportion of approximately 45–55% [11,12,21,23–28,30–35]. The duration of follow-up ranged from 3.5 to 18 years. Most studies (n = 10, 59%) reported a follow-up period of seven years or more [12,21,22,25,27–30,33,35].

Information on multimorbidity was typically collected through self-report [11,12,21,24–26,28,30,31,33,35], medical records or clinical examinations [29,32], or a combination of both [27,34]. Most studies used standardised scales to assess depression, mainly the Center for Epidemiologic Studies Depression Scale and Patient Health Questionnaire-9. Only three studies determined depressive status based on medical records or clinical diagnoses [29,32,33]. The number of chronic conditions included in the studies ranged from 7 to 69. This substantial variation in the number and types of conditions included may have contributed to differences in multimorbidity prevalence and estimated associations with depression across studies. Seven studies quantified the number of chronic conditions [12,22–26,35]. Eight studies used methods such as exploratory factor analysis or latent class analysis to identify multimorbidity patterns [21,22,25,28–32]. Four studies examined the association between multimorbidity and incident depression [11,27,33,34]. One study assessed trajectories of multimorbidity using a population-based trajectory model [35]. According to the Newcastle–Ottawa Scale, the overall quality of the included studies was high; 15 studies were rated as high quality, and 2 as moderate quality (Table S3 in the **Online Supplementary Document**).

### Multimorbidity and the risk of depression

Seven studies examined the association between multimorbidity and the risk of incident depression [12,22–26,35]. Four of these studies defined multimorbidity as the coexistence of ≥2 chronic conditions [12,23,27,35]. However, due to differences in reference groups and outcome measures, only descriptive analyses were conducted. Although there were some differences in the definitions of multimorbidity and stratification criteria across studies, all included studies identified a positive association between an increasing number of chronic conditions and the risk of depression. Du *et al*. provided further high-quality evidence from a multi-cohort study across six countries [12]. Compared with individuals without multimorbidity, those with multimorbidity had a significantly increased risk of depression (HR ≈ 1.30). The risk increased progressively with the number of chronic conditions, with the highest HR reaching approximately 1.57. Stratified analyses showed that this association was consistent across genders, with a slightly higher risk among men compared to women (HR ≈ 1.40 *vs.* HR ≈ 1.31).

**Figure 2:**
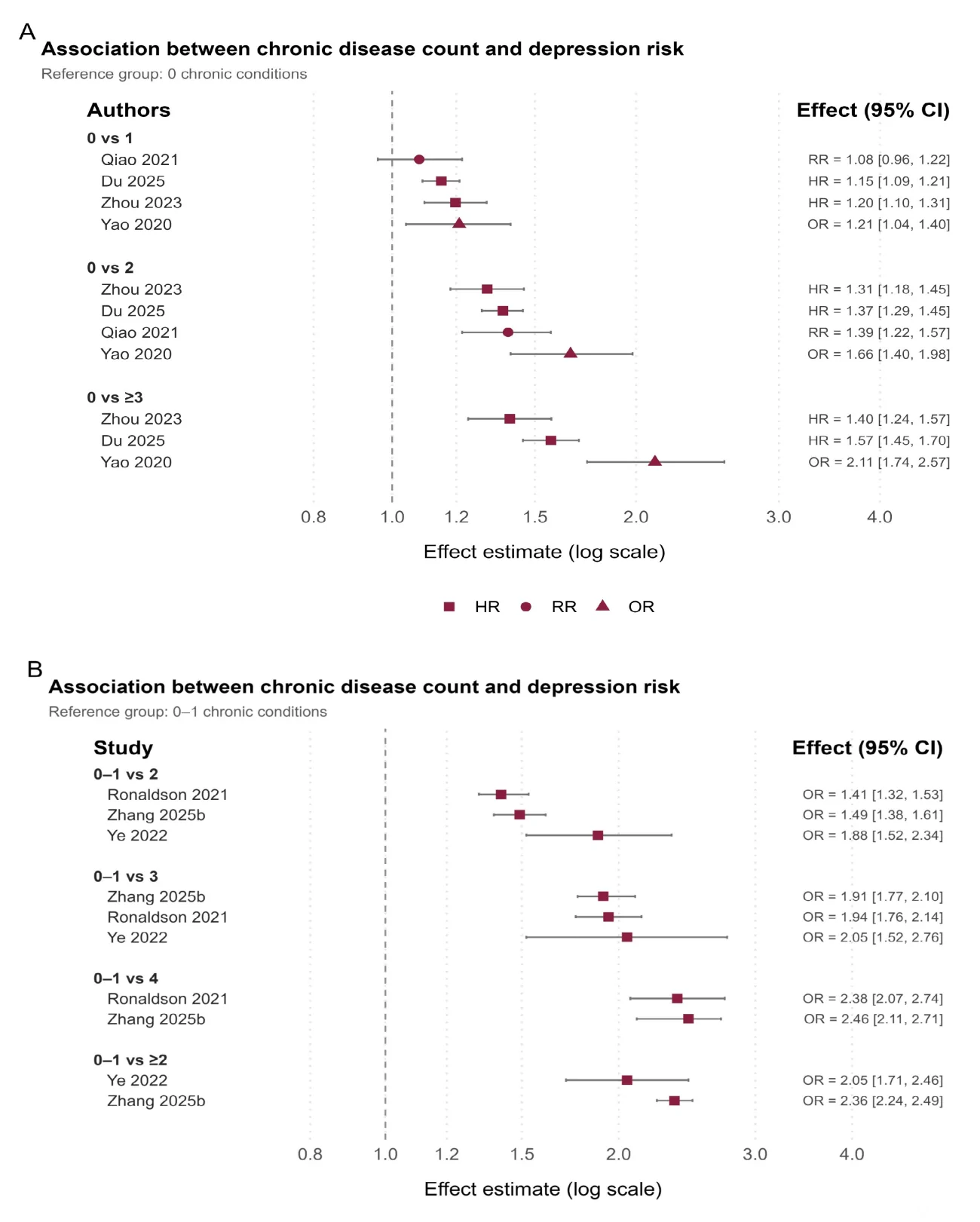
Association between the number of conditions and the risk of depression outcomes. **Panel A.** Reference group: 0 chronic conditions. **Panel B.** Reference group: 0–1 chronic conditions.

Regardless of whether the reference group was defined as individuals without chronic diseases (reference: 0) or those without multimorbidity (reference: 0–1), an increase in the number of chronic conditions was associated with a higher risk of incident depression (**Figure 2**, Panels A and B). A clear gradient effect was observed. With 0–1 chronic conditions as the reference group, greater multimorbidity was associated with an increased risk of incident depression, exhibiting a consistent dose-response pattern (**Figure 2**, Panel B). Although studies reported different effect measures and used different reference groups, the direction of the associations was generally consistent. Therefore, we used forest plots to provide a descriptive visual summary rather than a direct quantitative comparison of effect estimates.

### Multimorbidity patterns and the risk of depression

Eight studies examined the association between different comorbidity patterns and the risk of incident depression [21,22,25,28–32]. Of these, five studies identified comorbidity patterns using exploratory factor analysis or latent class analysis [22,25,28–30], while the remaining three were based on the prevalence of chronic diseases [31], predefined classifications of bodily systems (*e.g.* cardiovascular and musculoskeletal patterns) [31], and k-modes cluster analysis [12]. Differences in types of diseases included and the classification of patterns have led to significant heterogeneity and a degree of overlap in comorbidity patterns across studies. Following standardised integration of comorbidity patterns from each study, these can be broadly categorised into four main patterns: cardiovascular-metabolic, respiratory-related, musculoskeletal/inflammatory-related, and complex high comorbidity burden patterns. Most studies have observed an association between specific comorbidity patterns and an increased risk of depression. Among these, cardiovascular-metabolic and respiratory-related patterns were the most frequently studied and showed the most consistently reported associations with depression. Although there are differences in the strength of the association and the composition of the patterns across studies, other patterns involving gastrointestinal, musculoskeletal and pain-related conditions are also associated with an increased risk of depression.

**Figure 3:**
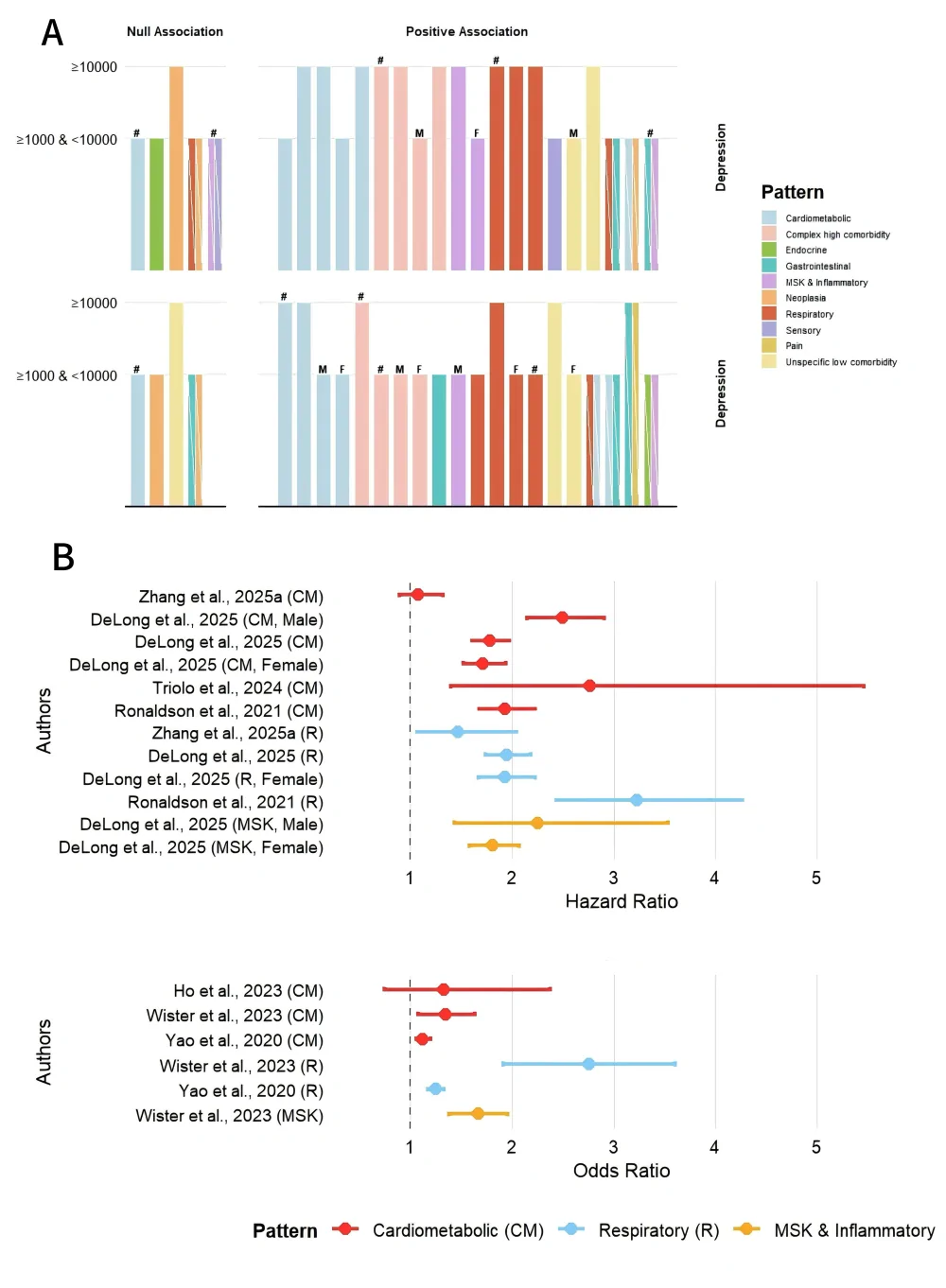
Associations between multimorbidity patterns and depression outcomes. **Panel A.** Harvest plot. **Panel B.** Forest plot. In Panel A, bar height reflects study sample size, and colours indicate the primary disease system in each pattern. In Panel B, the dashed vertical line indicates the null value. #Association was estimated using a non-specific multimorbidity pattern as the reference group. CM – cardiometabolic, F – female, M – male, MSK – musculoskeletal, R – respiratory.

Across the included studies, most multimorbidity patterns were positively associated with incident depression (**Figure 3**, Panel A). This finding was consistent across studies with different sample sizes. However, a small number of patterns showed weak or non-significant associations.

We summarised the major multimorbidity patterns and used a forest plot to illustrate their associations with the risk of depression (**Figure 3**, Panel B). The results showed that most multimorbidity patterns were associated with an increased risk of depression, although effect sizes varied and some associations were weak or non-significant. The respiratory pattern showed the highest effect size, with some studies reporting values of >3 [22]. The cardiovascular-metabolic pattern demonstrated a consistent positive association, with effect sizes mostly ranging between 1.3 and 2.5 [22,25,28–32]. The musculoskeletal or inflammatory pattern also showed a positive association, but with relatively lower effect sizes [31,32].

DeLong *et al*. found that all multimorbidity patterns showed consistent positive associations in both men and women, although the effect sizes differed [32]. Overall, the musculoskeletal-related pattern showed a relatively stronger effect in men, whereas the cardiovascular-metabolic pattern was more stable in women. The combined respiratory and chronic geriatric disease pattern in men (HR = 2.25; 95% CI = 1.43–3.53) showed a higher effect size than the respiratory-only pattern in women.

### Cardiometabolic multimorbidity and the risk of depression

Four studies examined the association between cardiovascular-metabolic multimorbidity and the risk of incident depression [11,27,33,34]. Two studies used the same set of conditions to define cardiovascular-metabolic multimorbidity [11,33]. One study additionally included hypertension and hyperlipidaemia [34] while another focused only on the cumulative effect of the number of cardiovascular-metabolic conditions [27]. Each cardiovascular or metabolic condition was associated with an increased risk of depression (Figure S1 in the **Online Supplementary Document**). The effect sizes for diabetes, heart disease, and stroke generally ranged between 1.1 and 1.7. As the number of cardiovascular-metabolic conditions increased, the risk of depression showed a gradual upward trend, suggesting a cumulative effect. However, results for specific disease combinations varied across studies. This variation may be related to differences in sample size and the number of conditions included. Zhou *et al.*, using a multinational cohort database from five countries, showed that individuals with cardiovascular-metabolic multimorbidity had a higher risk of depression (HR ≈ 1.40) than those without multimorbidity [11]. Different disease combinations showed varying levels of risk, but the findings were consistent across countries. Huang *et al.* reported that stroke, heart disease, and diabetes had clear independent effects [34]. In contrast, hypertension and dyslipidaemia were not significantly associated with depression when present alone. However, the risk increased markedly when these conditions co-occurred with other diseases. This suggests that they may act as background contributors within multimorbidity rather than independent risk factors.

### Multimorbidity trajectories and the risk of depression

Zhang *et al*. conducted a study using the CHARLS database, including 15,896 middle-aged and older participants [35]. The study examined the association between different multimorbidity trajectories and the risk of developing depression. After adjusting for relevant confounders, the results showed clear differences across trajectory groups. With the stable low multimorbidity group as the reference, the risk was the highest in the rapidly increasing group (RR = 4.40; 95% CI = 3.62–5.34), followed by the continuously increasing (RR = 2.54; 95% CI = 2.24–2.89) and slowly increasing group (RR = 1.53; 95% CI = 1.36–1.71). These findings suggest that dynamic changes in multimorbidity burden may be associated with the subsequent risk of depression.

## DISCUSSION

Based on evidence from cohort studies, we synthesised longitudinal associations between multimorbidity and depression outcomes. Overall, multimorbidity was generally associated with an increased risk of incident depression or depressive symptoms, and the risk tended to increase as the number of chronic conditions rose. The findings also suggest that the association may vary according to disease composition, with cardiovascular-metabolic and respiratory-related patterns showing the most consistently reported positive associations. However, these patterns were also among the most frequently investigated, and their apparent consistency may partially reflect the larger evidence base available for these categories. Evidence from one cohort study further suggested that increasing multimorbidity trajectories may be associated with subsequent depression risk [35], although this finding remains preliminary and requires replication in independent cohorts. Taken together, these findings indicate that the association between multimorbidity and depression may be related not only to disease burden, but also to disease composition and changes in multimorbidity over time.

The gradient relationship between the number of chronic conditions and depression risk suggests that multimorbidity may represent a precursor risk state with persistent effects rather than simply reflecting overall health vulnerability [36,37]. Multiple factors may explain this relationship. At the biological level, the coexistence of multiple chronic diseases is often accompanied by inflammatory responses, metabolic abnormalities, and neuroendocrine imbalances, which may affect emotional regulation [38–40]. At the functional level, a higher burden of multimorbidity is often associated with activity limitations and sleep disturbances. These factors may impair an individual’s ability to perform daily activities [41,42]. At the psychosocial level, long-term disease management and caregiving demands can lead to persistent chronic stress [43,44]. Taken together, the cumulative effect of these factors over time may contribute to the onset of depression.

Compared with classifications based solely on the number of chronic conditions, we observed clear differences in the associations between various multimorbidity patterns and the risk of depression. The cardiovascular-metabolic pattern showed one of the most consistently reported associations with depression across the included studies. This pattern typically includes conditions such as hypertension, diabetes, heart disease, and stroke. Its relationship with depression may involve multiple interrelated physiological processes. Although these mechanisms were not directly examined in the included studies, evidence from broader biological and epidemiological literature provides several plausible explanations for the observed associations. Vascular damage and insufficient cerebral perfusion may affect brain regions involved in emotional regulation [45], while chronic low-grade inflammation [46], insulin resistance, and metabolic disorders [47] may increase susceptibility to depression by disrupting neurotransmitter activity and brain function. Neuroendocrine dysfunction may further contribute to these processes [48], suggesting that their combined effects may be cumulative. As the number of coexisting conditions increases, the risk of depression appears to rise further. This suggests that different diseases may act synergistically within a multimorbidity context. In contrast, the impact of respiratory-related patterns is more evident at symptomatic and functional levels. Persistent symptoms such as dyspnoea, chronic cough, and nocturnal disturbances may impair sleep quality, daily functioning, and reduce social participation, thereby increasing the risk of depression [49,50]. These findings suggest that, in multimorbidity patterns characterized by a high burden of chronic conditions and functional limitations, symptom burden and functional impairment may be more directly associated with depression. The relatively large effect estimates reported for some respiratory-related patterns should be interpreted cautiously. These findings may reflect not only the substantial symptom burden associated with respiratory diseases, but also methodological differences across studies, including variation in pattern definitions, reference groups, and analytical approaches. Therefore, the observed associations require further confirmation in additional longitudinal cohorts. Musculoskeletal or inflammatory patterns are also associated with depression risk, but their mechanisms appear more concentrated. Their effects are mainly mediated through chronic pain and functional limitations [51]. Persistent pain may reduce functional capacity and, over time, affect emotional well-being [52]. In addition, inflammatory responses may mediate the relationship between pain and depression [53]. It should also be noted that evidence was not uniformly consistent across all multimorbidity patterns. Some patterns were supported by only a small number of studies, while several reported weak or statistically non-significant associations. Therefore, caution is warranted when interpreting differences across specific multimorbidity patterns. Overall, different multimorbidity patterns may be linked to depression through partly distinct and overlapping pathways, including increased physiological burden, functional limitations, and life stress. These mechanisms are presented as plausible explanations based on existing biological and epidemiological literature, and the proposed pathways should be interpreted as theoretical mechanisms requiring further investigation.

### Clinical implications

The findings of this study have important clinical and public health implications. First, in the management of chronic diseases, the burden and patterns of multimorbidity may be considered as part of frameworks for the early identification of depression risk. This approach moves beyond relying solely on passive screening after depressive symptoms have already developed. Second, for groups with potentially higher risk – such as individuals with cardiovascular-metabolic multimorbidity, respiratory-related multimorbidity, or rapidly increasing multimorbidity burden – more proactive mental health assessment and support may be considered in routine chronic disease follow-up, pending further validation. Third, the management of individuals with multimorbidity should not be limited to a single-disease approach. Greater attention should be given to functional status and mental health alongside physical conditions. This may improve the comprehensiveness of risk identification and intervention. Finally, identifying multimorbidity patterns associated with a higher risk of depression may help inform future risk stratification approaches. However, further standardisation and validation are required before pattern-specific clinical recommendations can be established.

### Strengths and limitations

This study has several limitations. First, the included studies showed considerable variation in the definition of multimorbidity, the number and scope of chronic conditions included, methods for pattern identification, and tools used to assess depression. The number of chronic conditions used to define multimorbidity ranged from 7 to 69, and the specific conditions included differed substantially across studies. These differences may have influenced both multimorbidity prevalence and the estimated associations with depression, thereby contributing to heterogeneity and limiting direct comparability and quantitative pooling of results. Second, depression outcomes were assessed using different approaches, including symptom screening scales, self-reported diagnoses, clinical diagnoses, and medical records. Differences in diagnostic thresholds and measurement methods may have contributed to heterogeneity across studies and should be considered when interpreting the findings. Third, differences in control group definitions and multimorbidity pattern classification further limited direct comparison across studies and precluded quantitative pooling of results. Fourth, evidence regarding multimorbidity trajectories was limited to a single cohort study and therefore requires confirmation in additional longitudinal populations. Finally, although cohort studies help clarify temporal relationships, residual confounding and potential reverse associations cannot be fully ruled out. Publication bias cannot be excluded, as studies reporting statistically significant associations may be more likely to be published than studies reporting null findings. Moreover, several included studies were derived from the same large cohort databases, particularly CHARLS. While these studies addressed different research questions and examined distinct multimorbidity measures, patterns, or trajectories, overlap in source populations may have contributed to the apparent consistency of some findings.

## CONCLUSIONS

Evidence from longitudinal cohort studies indicates that multimorbidity is consistently associated with an increased risk of incident depression or depressive symptoms. This association is reflected not only in the number of chronic conditions, but also in specific multimorbidity patterns and their dynamic trajectories. Cardiovascular-metabolic patterns, respiratory-related patterns, and rapidly increasing trajectories of multimorbidity may represent potential high-risk features that warrant further validation. We synthesised current longitudinal evidence regarding the relationship between multimorbidity and depression at both the level of disease count and multimorbidity patterns. Future research should standardise definitions of multimorbidity and methods for pattern identification. It should also strengthen investigations into the dynamic processes of multimorbidity and its underlying mechanisms. This may provide a stronger evidence base for future research on early identification, risk stratification, and intervention development for depression among individuals with multimorbidity.

## Additional material


Online Supplementary Document
