# Supplementary material for: Multimorbidity and the risk of depression: a systematic review of cohort studies
Source: J Glob Health. 2026 Sep 25;16:04279. doi: 10.7189/jogh.16.04279 (PMC13613292; doi:10.7189/jogh.16.04279)
Supplement: Supplementary file 1 [file jogh-16-04279-s001.pdf]

Supplement to: Wu J, Xu K, Jiang P, Mei H. Multimorbidity and the risk of depression: a systematic review of cohort studies. J Glob Health. 2026;16:04279.

Table S1 PRISMA 2020 checklist

Section and Topic	Item #	Checklist item	Location where item is reported
**TITLE**			
Title	1	Identify the report as a systematic review.	Page 1
**ABSTRACT**			
Abstract	2	See the PRISMA 2020 for Abstracts checklist.	Page 1
**INTRODUCTION**			
Rationale	3	Describe the rationale for the review in the context of existing knowledge.	Page 2-4
Objectives	4	Provide an explicit statement of the objective(s) or question(s) the review addresses.	Page 3-4
**METHODS**			
Eligibility criteria	5	Specify the inclusion and exclusion criteria for the review and how studies were grouped for the syntheses.	Page 4-6
Information sources	6	Specify all databases, registers, websites, organisations, reference lists and other sources searched or consulted to identify studies. Specify the date when each source was last searched or consulted.	Page 4
Search strategy	7	Present the full search strategies for all databases, registers and websites, including any filters and limits used.	Supplementary material S2
Selection process	8	Specify the methods used to decide whether a study met the inclusion criteria of the review, including how many reviewers screened each record and each report retrieved, whether they worked independently, and if applicable, details of automation tools used in the process.	Page 5-6
Data collection process	9	Specify the methods used to collect data from reports, including how many reviewers collected data from each report, whether they worked independently, any processes for obtaining or confirming data from study investigators, and if applicable, details of automation tools used in the process.	Page 5-6
Data items	10a	List and define all outcomes for which data were sought. Specify whether all results that were compatible with each outcome domain in each study were sought (e.g. for all measures, time points, analyses), and if not, the methods used to decide which results to collect.	Page 5-6
	10b	List and define all other variables for which data were sought (e.g. participant and intervention characteristics, funding sources). Describe any assumptions made about any missing or unclear information.	NR
Study risk of bias assessment	11	Specify the methods used to assess risk of bias in the included studies, including details of the tool(s) used, how many reviewers assessed each study and whether they worked independently, and if applicable, details of automation tools used in the process.	Page 5
Effect measures	12	Specify for each outcome the effect measure(s) (e.g. risk ratio, mean difference) used in the synthesis or presentation of results.	Table 1
Synthesis metho	13a	Describe the processes used to decide which studies were eligible for e	Figure 1

Section and Topic	Item #	Checklist item	Location where item is reported
ds		ach synthesis (e.g. tabulating the study intervention characteristics and comparing against the planned groups for each synthesis (item #5)).	
	13b	Describe any methods required to prepare the data for presentation or synthesis, such as handling of missing summary statistics, or data conversions.	Page 5
	13c	Describe any methods used to tabulate or visually display results of individual studies and syntheses.	Page 5-6
	13d	Describe any methods used to synthesize results and provide a rationale for the choice(s). If meta-analysis was performed, describe the model(s), method(s) to identify the presence and extent of statistical heterogeneity, and software package(s) used.	Page 5-6
	13e	Describe any methods used to explore possible causes of heterogeneity among study results (e.g. subgroup analysis, meta-regression).	NR
	13f	Describe any sensitivity analyses conducted to assess robustness of the synthesized results.	NR
Reporting bias assessment	14	Describe any methods used to assess risk of bias due to missing results in a synthesis (arising from reporting biases).	NR
Certainty assessment	15	Describe any methods used to assess certainty (or confidence) in the body of evidence for an outcome.	NR; rationale provided in Methods Section 2.5 and Limitations
**RESULTS**			
Study selection	16a	Describe the results of the search and selection process, from the number of records identified in the search to the number of studies included in the review, ideally using a flow diagram.	Figure 1
	16b	Cite studies that might appear to meet the inclusion criteria, but which were excluded, and explain why they were excluded.	Figure 1
Study characteristics	17	Cite each included study and present its characteristics.	Table 1
Risk of bias in studies	18	Present assessments of risk of bias for each included study.	Supplementary material S3
Results of individual studies	19	For all outcomes, present, for each study: (a) summary statistics for each group (where appropriate) and (b) an effect estimate and its precision (e.g. confidence/credible interval), ideally using structured tables or plots.	Table 1
Results of syntheses	20a	For each synthesis, briefly summarise the characteristics and risk of bias among contributing studies.	Page 6
	20b	Present results of all statistical syntheses conducted. If meta-analysis was done, present for each the summary estimate and its precision (e.g. confidence/credible interval) and measures of statistical heterogeneity. If comparing groups, describe the direction of the effect.	Page 6-9
	20c	Present results of all investigations of possible causes of heterogeneity among study results.	NR
	20d	Present results of all sensitivity analyses conducted to assess the robustness of the synthesized results.	NR
Reporting biases	21	Present assessments of risk of bias due to missing results (arising from reporting biases) for each synthesis assessed.	NR
Certainty of evidence	22	Present assessments of certainty (or confidence) in the body of evidence for each outcome assessed.	NR

Section and Topic	Item #	Checklist item	Location where item is reported
DISCUSSION			
Discussion	23a	Provide a general interpretation of the results in the context of other evidence.	Page 9-10
	23b	Discuss any limitations of the evidence included in the review.	Page 11
	23c	Discuss any limitations of the review processes used.	Page 11
	23d	Discuss implications of the results for practice, policy, and future research.	Page 10
OTHER INFORMATION			
Registration and protocol	24a	Provide registration information for the review, including register name and registration number, or state that the review was not registered.	Page 4
	24b	Indicate where the review protocol can be accessed, or state that a protocol was not prepared.	Page 4
	24c	Describe and explain any amendments to information provided at registration or in the protocol.	NR
Support	25	Describe sources of financial or non-financial support for the review, and the role of the funders or sponsors in the review.	Page 12
Competing interests	26	Declare any competing interests of review authors.	Page 12
Availability of data, code and other materials	27	Report which of the following are publicly available and where they can be found: template data collection forms; data extracted from included studies; data used for all analyses; analytic code; any other materials used in the review.	Page 12

From: Page MJ, McKenzie JE, Bossuyt PM, Boutron I, Hoffmann TC, Mulrow CD, et al. The PRISMA 2020 statement: an updated guideline for reporting systematic reviews. BMJ 2021;372:n71. doi: 10.1136/bmj.n7

Table S2 Full search strategy

Final search date for all databases: 5 March 2026.

PubMed search strategy.

No.	Query	Results
#1	"Multimorbidity"[MeSH Terms] OR "Comorbidity"[MeSH Terms]	137660
#2	"comorbidities"[Title/Abstract] OR "multi-morbidity"[Title/Abstract] OR "chronic disease"[Title/Abstract] OR "multiple chronic conditions"[Title/Abstract] OR "multiple chronic diseases"[Title/Abstract] OR "multipathology"[Title/Abstract] OR "multimorbidities"[Title/Abstract] OR "multiple long term conditions"[Title/Abstract] OR "multiple morbidity"[Title/Abstract] OR "multiple morbidities"[Title/Abstract] OR "coexisting condition"[Title/Abstract] OR "co existing disease"[Title/Abstract] OR (("cooccur"[All Fields] OR "cooccurred"[All Fields] OR "cooccurring"[All Fields]) AND "condition"[Title/Abstract]) OR "concurrent condition"[Title/Abstract] OR "multiple diseases"[Title/Abstract] OR "associated disease"[Title/Abstract] OR "cardiometabolic multimorbidity"[Title/Abstract] OR "cardio-metabolic"[Title/Abstract] OR "cardiometabolic"[Title/Abstract] OR "psychosomatic comorbidity"[Title/Abstract] OR "physical mental comorbidity"[Title/Abstract] OR "somatic psychiatric comorbidity"[Title/Abstract] OR "multimorbidity pattern"[Title/Abstract] OR "multimorbidity cluster"[Title/Abstract] OR "chronic disease cluster"[Title/Abstract]	295421

#3	#1 OR #2	398673
#4	"depressive disorder"[MeSH Terms] OR "depression"[MeSH Terms]	292068
#5	"depress"[Title/Abstract] OR "depressive disorders"[Title/Abstract] OR "emotional depression"[Title/Abstract] OR "affective disorder"[Title/Abstract] OR "mood disorders"[Title/Abstract] OR "mental disorders"[Title/Abstract] OR "dysthymic disorder"[Title/Abstract] OR "Melancholia"[Title/Abstract] OR "major depressive disorder"[Title/Abstract] OR "major depressive disorders"[Title/Abstract] OR "clinical depression"[Title/Abstract] OR "depressions clinical"[Title/Abstract] OR "bipolar disorder"[Title/Abstract] OR "bipolar depression"[Title/Abstract] OR "depressive symptom"[Title/Abstract] OR "incident depression"[Title/Abstract] OR "new onset depression"[Title/Abstract] OR "depressive episode"[Title/Abstract]	183397
#6	#4 OR #5	412954
#7	"cohort studies"[MeSH Terms] OR "longitudinal studies"[MeSH Terms]	2856127
#8	"cohort studies"[Title/Abstract] OR "longitudinal studies"[Title/Abstract] OR "follow up studies"[Title/Abstract] OR "prospective studies"[Title/Abstract] OR "retrospective studies"[Title/Abstract] OR "incidence studies"[Title/Abstract] OR ((("historic"[All Fields] OR "historical"[All Fields] OR "historically"[All Fields] OR "historicity"[All Fields]) AND ("Cohort"[All Fields] OR "cohort s"[All Fields] OR "cohorte"[All Fields] OR "cohorts"[All Fields])) AND "incidence studies"[Title/Abstract]) OR "cohort analysis"[Title/Abstract] OR "prospective analysis"[Title/Abstract] OR "longitudinal analysis"[Title/Abstract] OR "concurrent study"[Title/Abstract]	218245
#9	#7 OR #8	2979680
#10	#3 AND #6 AND #9	8000
#11	"humans"[MeSH Terms] AND "English"[Language] AND #10	7736
#12	#11 AND (1000/1/1:2025/12/31[pdat])	7698

Web of Science search strategy.

No.	Query	Results
#1	AB=("Multimorbidity" OR "Comorbidity" OR "comorbidities" OR "multi-morbidity" OR "Chronic Disease" OR "Multiple Chronic Conditions" OR "multiple chronic diseases" OR "multimorbidities" OR "Multiple Long-Term Conditions" OR "Multiple Morbidity" OR "Multiple Morbidities" OR "coexisting condition" OR "co-existing disease" OR "cooccurring condition" OR "concurrent condition" OR "multiple diseases" OR "associated disease" OR "cardiometabolic multimorbidity" OR "cardio-metabolic" OR "cardiometabolic" OR "Psychosomatic Comorbidity" OR "Physical-Mental Comorbidity" OR "Somatic-Psychiatric Comorbidity" OR "multimorbidity pattern" OR "multimorbidity cluster" OR "chronic disease cluster")	235232
#2	TI=("Multimorbidity" OR "Comorbidity" OR "comorbidities" OR "multi-morbidity" OR "Chronic Disease" OR "Multiple Chronic Conditions" OR "multiple chronic diseases" OR "multimorbidities" OR "Multiple Long-Term Conditions" OR "Multiple Morbidity" OR "Multiple Morbidities" OR "coexisting condition" OR "co-existing disease" OR "cooccurring condition" OR "concurrent condition	50128

	" OR "multiple diseases" OR "associated disease" OR "cardiometabolic multimorbidity" OR "cardio-metabolic" OR "cardiometabolic" OR "Psychosomatic Comorbidity" OR "Physical-Mental Comorbidity" OR "Somatic-Psychiatric Comorbidity" OR "multimorbidity pattern" OR "multimorbidity cluster" OR "chronic disease cluster")	
#3	#1 OR #2	257771
#4	AB=("Depression" OR "Depressive Disorder" OR "depress" OR "depressive disorders" OR "Emotional Depression" OR "Affective Disorder" OR "Mood Disorders" OR "Mental Disorders" OR "Dysthymic Disorder" OR "Melancholia" OR "Major Depressive Disorder" OR "Major Depressive Disorders" OR "Clinical Depression" OR "Depressions Clinical" OR "Bipolar Disorder" OR "Bipolar Depression" OR "depressive symptom" OR "incident depression" OR "new-onset depression" OR "depressive episode")	311421
#5	TI=("Depression" OR "Depressive Disorder" OR "depress" OR "depressive disorders" OR "Emotional Depression" OR "Affective Disorder" OR "Mood Disorders" OR "Mental Disorders" OR "Dysthymic Disorder" OR "Melancholia" OR "Major Depressive Disorder" OR "Major Depressive Disorders" OR "Clinical Depression" OR "Depressions Clinical" OR "Bipolar Disorder" OR "Bipolar Depression" OR "depressive symptom" OR "incident depression" OR "new-onset depression" OR "depressive episode")	146393
#6	#4 OR #5	361420
#7	AB=("Cohort Study" OR "Longitudinal Study" OR "Cohort Studies" OR "Longitudinal Studies" OR "follow-up Studies" OR "Prospective Studies" OR "Retrospective Studies" OR "Incidence Studies" OR "Historical Cohort Incidence Studies" OR "Cohort Analysis" OR "prospective analysis" OR "longitudinal analysis" OR "Concurrent Study")	394109
#8	TI=("Cohort Study" OR "Longitudinal Study" OR "Cohort Studies" OR "Longitudinal Studies" OR "follow-up Studies" OR "Prospective Studies" OR "Retrospective Studies" OR "Incidence Studies" OR "Historical Cohort Incidence Studies" OR "Cohort Analysis" OR "prospective analysis" OR "longitudinal analysis" OR "Concurrent Study")	188205
#9	#7 OR #8	508833
#10	#3 AND #6 AND #9	3071
#11	#10 AND LA=(English)	3045
#12	#11and 2026 (Exclude – Publication Years)	2984

Embase search strategy

No.	Query	Results
#1	'comorbidity'/exp OR 'multiple chronic conditions'/exp	530099
#2	comorbidities:ab,ti OR 'multi morbidity':ab,ti OR 'chronic disease':ab,ti OR 'multiple chronic conditions':ab,ti OR 'multiple chronic diseases':ab,ti OR multipathology:ab,ti OR multimorbidities:ab,ti OR 'multiple long-term conditions':ab,ti OR 'multiple morbidity':ab,ti OR 'multiple morbidities':ab,ti OR 'coexisting condition':ab,ti OR 'co-existing disease':ab,ti OR 'cooccurring condition':ab,ti OR 'concurrent condition':ab,ti OR 'multiple diseases':ab,ti OR 'associated disease':ab,ti OR 'cardiometabolic multimorbidity':ab,ti OR '	501001

	cardio metabolic':ab,ti OR cardiometabolic:ab,ti OR 'psychosomatic comorbidity':ab,ti OR 'physical-mental comorbidity':ab,ti OR 'somatic-psychiatric comorbidity':ab,ti OR 'multimorbidity pattern':ab,ti OR 'multimorbidity cluster':ab,ti OR 'chronic disease cluster':ab,ti	
#3	#1 OR #2	788578
#4	'depression'/exp	820737
#5	depress:ab,ti OR 'depressive disorders':ab,ti OR 'emotional depression':ab,ti OR 'affective disorder':ab,ti OR 'mood disorders':ab,ti OR 'mental disorders':ab,ti OR 'dysthymic disorder':ab,ti OR melancholia:ab,ti OR 'major depressive disorder':ab,ti OR 'major depressive disorders':ab,ti OR 'clinical depression':ab,ti OR 'depressions clinical':ab,ti OR 'bipolar disorder':ab,ti OR 'bipolar depression':ab,ti OR 'depressive symptom':ab,ti OR 'incident depression':ab,ti OR 'new-onset depression':ab,ti OR 'depressive episode':ab,ti	239903
#6	#4 OR #5	893926
#7	'cohort analysis'/exp OR 'longitudinal study'/exp	1777935
#8	'longitudinal studies':ab,ti OR 'cohort studies':ab,ti OR 'follow-up studies':ab,ti OR 'prospective studies':ab,ti OR 'retrospective studies':ab,ti OR 'incidence studies':ab,ti OR 'historical cohort incidence studies':ab,ti OR 'cohort analysis':ab,ti OR 'prospective analysis':ab,ti OR 'longitudinal analysis':ab,ti OR 'concurrent study':ab,ti	301281
#9	#7 OR #8	1966690
#10	#3 AND #6 AND #9	15456
#11	#10 AND english:la	15343
#12	#11 AND (1970:py OR 1991:py OR 1992:py OR 1994:py OR 1995:py OR 1996:py OR 1997:py OR 1998:py OR 1999:py OR 2000:py OR 2001:py OR 2002:py OR 2003:py OR 2004:py OR 2005:py OR 2006:py OR 2007:py OR 2008:py OR 2009:py OR 2010:py OR 2011:py OR 2012:py OR 2013:py OR 2014:py OR 2015:py OR 2016:py OR 2017:py OR 2018:py OR 2019:py OR 2020:py OR 2021:py OR 2022:py OR 2023:py OR 2024:py OR 2025:py)	14908

APA psycInfo search strategy

No.	Query	Results
#1	TI ("Multimorbidity" OR "Comorbidity" OR "comorbidities" OR "multi-morbidity" OR "Chronic Disease" OR "Multiple Chronic Conditions" OR "multiple chronic diseases" OR "multimorbidities" OR "Multiple Long-Term Conditions" OR "Multiple Morbidity" OR "Multiple Morbidities" OR "coexisting condition" OR "co-existing disease" OR "cooccurring condition" OR "concurrent condition" OR "multiple diseases" OR "associated disease" OR "cardiometabolic multimorbidity" OR "cardio-metabolic" OR "cardiometabolic" OR "Psychosomatic Comorbidity" OR "Physical-Mental Comorbidity" OR "Somatic-Psychiatric Comorbidity" OR "multimorbidity pattern" OR "multimorbidity cluster" OR "chronic disease cluster") OR AB ("Multimorbidity" OR "Comorbidity" OR "comorbidities" OR "multi-morbidity" OR "Chronic Disease" OR "Multiple Chronic Conditions" OR "multiple chronic diseases" OR "multimorbidities" OR "Multiple Long-Term Conditions" OR "Multiple Morbidity" OR "Multiple Morbidities" OR "coexisting condition" OR "co-existing disease" OR "cooccurring condition" OR "concurrent con	57736

	dition" OR "multiple diseases" OR "associated disease" OR "cardiometabolic multimorbidity" OR "cardio-metabolic" OR "cardiometabolic" OR "Psychosomatic Comorbidity" OR "Physical-Mental Comorbidity" OR "Somatic-Psychiatric Comorbidity" OR "multimorbidity pattern" OR "multimorbidity cluster" OR "chronic disease cluster")	
#2	TI ("Depression" OR "Depressive Disorder" OR "depress" OR "depressive disorders" OR "Emotional Depression" OR "Affective Disorder" OR "Mood Disorders" OR "Mental Disorders" OR "Dysthymic Disorder" OR "Melancholia" OR "Major Depressive Disorder" OR "Major Depressive Disorders" OR "Clinical Depression" OR "Depressions Clinical" OR "Bipolar Disorder" OR "Bipolar Depression" OR "depressive symptom" OR "incident depression" OR "new-onset depression" OR "depressive episode") OR AB ("Depression" OR "Depressive Disorder" OR "depress" OR "depressive disorders" OR "Emotional Depression" OR "Affective Disorder" OR "Mood Disorders" OR "Mental Disorders" OR "Dysthymic Disorder" OR "Melancholia" OR "Major Depressive Disorder" OR "Major Depressive Disorders" OR "Clinical Depression" OR "Depressions Clinical" OR "Bipolar Disorder" OR "Bipolar Depression" OR "depressive symptom" OR "incident depression" OR "new-onset depression" OR "depressive episode")	399992
#3	TI ("Cohort Study" OR "Longitudinal Study" OR "Cohort Studies" OR "Longitudinal Studies" OR "follow-up Studies" OR "Prospective Studies" OR "Retrospective Studies" OR "Incidence Studies" OR "Historical Cohort Incidence Studies" OR "Cohort Analysis" OR "prospective analysis" OR "longitudinal analysis" OR "Concurrent Study") OR AB ("Cohort Study" OR "Longitudinal Study" OR "Cohort Studies" OR "Longitudinal Studies" OR "follow-up Studies" OR "Prospective Studies" OR "Retrospective Studies" OR "Incidence Studies" OR "Historical Cohort Incidence Studies" OR "Cohort Analysis" OR "prospective analysis" OR "longitudinal analysis" OR "Concurrent Study") OR PT (longitudinal studies)	112222
#4	#1 AND #2 AND #3	1657
#5	#4 and "language:english"	1603
#6	#5 and "date2025/12"	1546

CKNI

NO.	Query	Results
#1	(Topic:Comorbidity OR Multiple diseases OR Coexisting diseases OR Multiple chronic conditions OR Comorbidity patterns OR Coexisting chronic diseases OR Chronic disease comorbidity OR Multiple chronic diseases OR Chronic disease combinations OR Chronic disease overlap OR Disease comorbidity OR Multiple disease patterns OR Comorbidity clusters OR Cardiovascular-metabolic comorbidity OR Cardiac-metabolic multiple diseases OR Physical-psychological comorbidity OR Multisystem chronic diseases)	77,299
#2	**(Topic:Depression OR Major Depressive Disorder OR Major Depressive Disorder OR Mood Disorder OR Onset of Depression OR New-Onset Depression OR Depression Risk OR Occurrence of Depression OR Depressive Mood OR**	689,378

	Symptoms of Depression OR Depressive State OR Depressive Episode OR Anxiety and Depression OR Hamilton Depression Rating Scale OR Severe Mental Illness OR Major Depressive Disorder OR Mood Disorder)	
#3	(Topic:Cohort study OR prospective study OR longitudinal study OR follow-up study OR retrospective cohort OR long-term follow-up OR multicentre cohort study)	628,432
#4	#1 and #2 and #3	1298
#1	#4 and (Time range to December 31, 2025)	1275

Wanfang Database

NO.	Query	Results
#1	Topic:(Comorbidity OR Multiple diseases OR Coexisting diseases OR Multiple chronic conditions OR Comorbidity patterns OR Coexisting chronic diseases OR Chronic disease comorbidity OR Multiple chronic diseases OR Chronic disease combinations OR Chronic disease overlap OR Disease comorbidity OR Multiple disease patterns OR Comorbidity clusters OR Cardiovascular-metabolic comorbidity OR Cardiac-metabolic multiple diseases OR Physical-psychological comorbidity OR Multisystem chronic diseases)	174,740
#2	(Topic:Depression OR Major Depressive Disorder OR Major Depressive Disorder OR Mood Disorder OR Onset of Depression OR New-Onset Depression OR Depression Risk OR Occurrence of Depression OR Depressive Mood OR Symptoms of Depression OR Depressive State OR Depressive Episode OR Anxiety and Depression OR Hamilton Depression Rating Scale OR Severe Mental Illness OR Major Depressive Disorder OR Mood Disorder)	877,242
#3	(Topic:Cohort study OR prospective study OR longitudinal study OR follow-up study OR retrospective cohort OR long-term follow-up OR multicentre cohort study)	259,994
#4	#1 and #2 and #3	334
#1	#4 and (Time range to 2025)	333

Table S3 Risk of bias assessment according to NOS

Author (Year)	Selection				Comparability		Exposure			Scores	Quality
	Q1	Q2	Q3	Q4	Q5	Q6	Q7	Q8	Q9		
Hsu 2013	-	*	*	*	*	-	-	*	*	6	Fair
Yao 2020	*	-	*	*	*	*	-	*	*	7	High
Ronaldson2021	*	*	*	*	*	*	*	*	-	8	High
Qiao 2021	*	-	*	*	*	*	-	*	*	7	High
Ye 2022	*	*	*	*	*	-	-	*	*	7	High
Huang 2022	-	*	*	*	*	*	-	*	*	7	High
Ho 2023	-	*	*	*	*	*	-	*	-	6	Fair
Wister2023	*	*	*	*	*	*	-	*	*	8	High
Triolo 2024	*	*	*	*	*	*	*	*	*	9	High
Zhou 2023.	*	-	*	*	*	*	*	*	*	8	High

Yang 2024	-	*	*	*	*	*	*	*	*	8	High
Zhou 2024	*	*	*	*	*	*	-	*	*	8	High
Zhang2025a	*	*	*	*	*	*	-	*	*	8	High
Zhang2025b	*	*	-	*	*	*	-	*	*	7	High
DeLong2025	*	*	*	*	*	-	*	*	*	8	High
Guo 2025	*	-	*	*	*	*	-	*	*	7	High
Du2025	*	*	*	*	*	*	-	*	*	8	High

The NOS accredits one star for each subitem within the three categories.

<u>Selection</u>

Q1. Representativeness of the exposed cohort
Q2. Selection of the non-exposed cohort
Q3. Ascertainment of exposure
Q4. Demonstration that outcome of interest was not present at start of study

<u>Comparability</u>

Q5. The study controls for age, sex and marital status
Q6. Study controls for other factors

<u>Outcome</u>

Q7. Assessment of outcome
Q8. Was follow-up long enough for outcomes to occur
Q9. Adequacy of follow-up of cohorts

Table S4 **Conversion of colours coding charactering diseases for each multimorbidity patterns presented in the harvest plot (Figure. 3A)**

Hsu et al, 2013	1. Chronic respiratory disease<br>2. Cardiovascular disease<br>3. Gastrointestinal disease<br>4. Cancer<br>5. CRD + CVD<br>6. CRD + GI<br>7. CRD + cancer<br>8. CVD + GI<br>9. CVD + cancer<br>10. GI + cancer	1. Respiratory<br>2. Cardiometabolic<br>3. Gastrointestinal<br>4. Neoplasia<br>5. Respiratory AND Cardiometabol<br>6. Respiratory AND Gastrointesti<br>7. Respiratory AND Neoplasia<br>8. Cardiometabolic AND Gastrointes<br>9. Cardiometabolic AND Neoplasia<br>10. Gastrointestinal AND Neoplasi
Yao et al, 2020	1. Cardio-Metabolic<br>2. Respiratory<br>3. Arthritic-Digestive-Visual<br>4. Hepatic-Renal-Skeletal	1. Cardiometabolic<br>2. Respiratory<br>3. Complex high comorbidity<br>4. Complex high comorbidity
Ronaldson et al, 2021	1. Undefined multimorbidity<br>2. Cardiometabolic<br>3. Respiratory<br>4. Cardio/cerebrovascular<br>5. Reproductive<br>6. Pain/gastrointestinal	1. Unspecific low comorbidity<br>2. Cardiometabolic<br>3. Respiratory<br>4. Cardiometabolic<br>5. Unspecific low comorbidity<br>6. Gastrointestinal AND Pain
Triolo et al, 2024	1. Unspecific<br>2. Metabolic<br>3. Sensory/anaemia<br>4. Thyroid/musculoskeletal	1. Unspecific low comorbidity<br>2. Endocrine<br>3. Sensory<br>4. Endocrine AND MSK & Inflamma

	5. Cardiometabolic	5. Cardiometabolic
Wister et al, 2023	1. Cardio<br>2. Osteo<br>3. Respiratory<br>4. Cancer<br>5. Multiple<br>6. Other	1. Cardiometabolic<br>2. MSK & Inflammatory<br>3. Respiratory<br>4. Neoplasia<br>5. Complex high comorbidity<br>6. Unspecific low comorbidity
Ho et al, 2023	1. Cardiometabolic<br>2. Arthritis-cataract<br>3. Multimorbidity	1. Cardiometabolic<br>2. MSK & Inflammatory AND Senso<br>3. Complex high comorbidity
Zhang et al, 2025	1. Metabolic syndrome/vascular<br>2. Digestion/arthritis<br>3. Respiratory	1. Cardiomet<br>2. Gastrointestinal AND MS flammatory<br>3. Respiratory
DeLong et al, 2025	Overall:<br>1. Respiratory<br>2. CVD + Diabetes<br>3. Mixed including cancer<br>4. Healthy + Rhinitis<br>Women:<br>1. Respiratory<br>2. Musculoskeletal<br>3. CVD + Diabetes<br>4. Mixed including cancer<br>5. Healthy + Rhinitis<br>Men:<br>1. Musculoskeletal + others<br>2. Respiratory + gout + male genitourinary<br>3. CVD + Diabetes<br>4. Mixed including cancer<br>5. Healthy + Rhinitis	Overall:<br>1. Respi<br>2. Cardiomet<br>3. Complex high comor<br>4. Unspecific low comorb<br>Woman:<br>1. Respiratory<br>2. MSK & Inflammatory<br>3. Cardiometabolic<br>4. Complex high comorbidity<br>5. Unspecific low comorbidity<br>Men:<br>1. MSK & Inflammatory<br>2. Complex high comorbidity<br>3. Cardiometabolic<br>4. Complex high comorbidity<br>5. Unspecific low comorbidity

Figure S1 Association between a cluster of cardiovascular and metabolic diseases and the risk of depression outcomes.

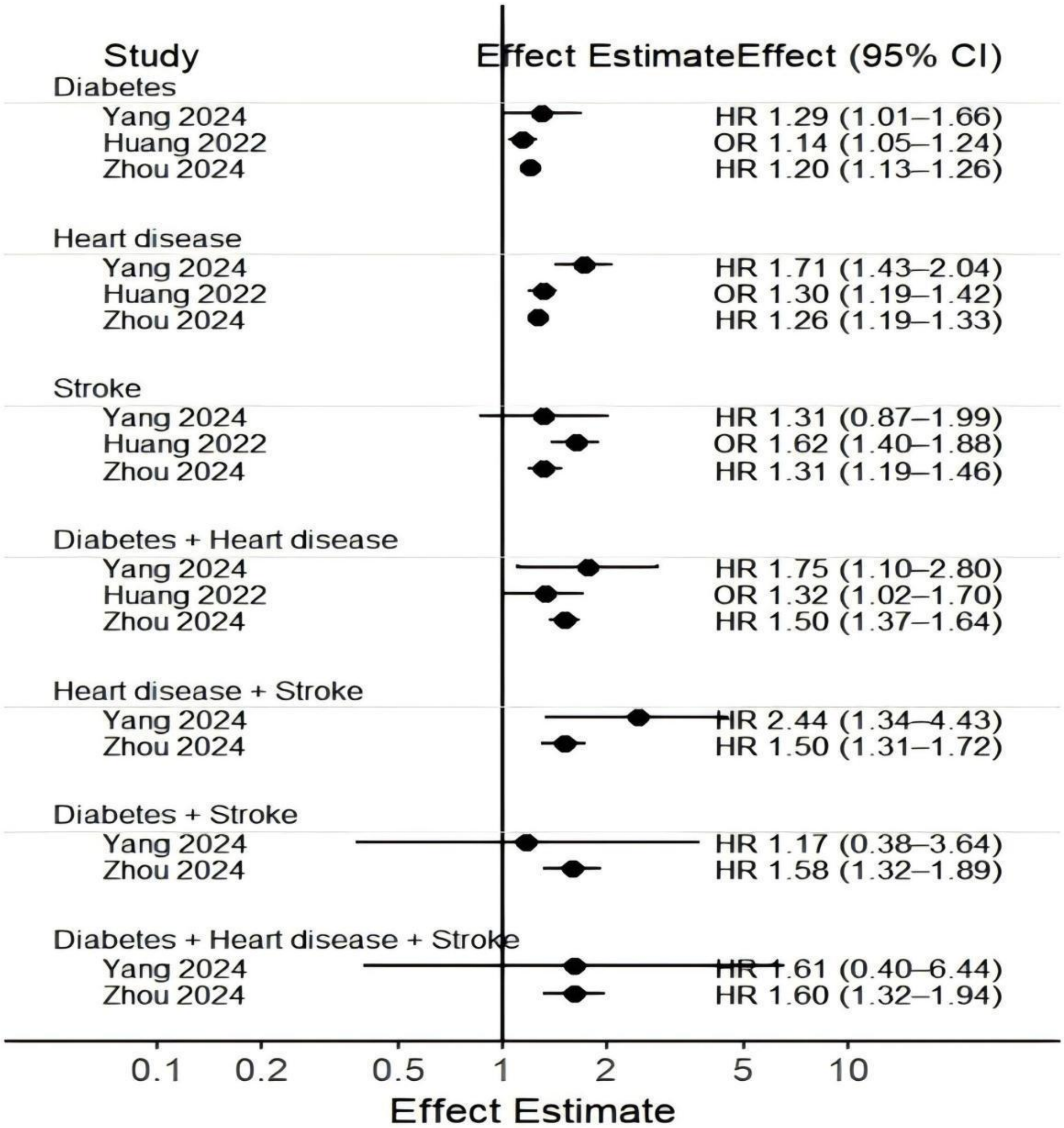

Study
Effect Estimate
Effect (95% CI)
Diabetes
Yang 2024 HR 1.29 (1.01–1.66)
Huang 2022 OR 1.14 (1.05–1.24)
Zhou 2024 HR 1.20 (1.13–1.26)
Heart disease
Yang 2024 HR 1.71 (1.43–2.04)
Huang 2022 OR 1.30 (1.19–1.42)
Zhou 2024 HR 1.26 (1.19–1.33)
Stroke
Yang 2024 HR 1.31 (0.87–1.99)
Huang 2022 OR 1.62 (1.40–1.88)
Zhou 2024 HR 1.31 (1.19–1.46)
Diabetes + Heart disease
Yang 2024 HR 1.75 (1.10–2.80)
Huang 2022 OR 1.32 (1.02–1.70)
Zhou 2024 HR 1.50 (1.37–1.64)
Heart disease + Stroke
Yang 2024 HR 2.44 (1.34–4.43)
Zhou 2024 HR 1.50 (1.31–1.72)
Diabetes + Stroke
Yang 2024 HR 1.17 (0.38–3.64)
Zhou 2024 HR 1.58 (1.32–1.89)
Diabetes + Heart disease + Stroke
Yang 2024 HR 1.61 (0.40–6.44)
Zhou 2024 HR 1.60 (1.32–1.94)
0.1
0.2
0.5
1
2
5
10
Effect Estimate
